# Sex, Resilience and Psychological Well-Being in Mexican University Students

**DOI:** 10.3390/ejihpe15030032

**Published:** 2025-03-10

**Authors:** Martha Ornelas, Perla Jannet Jurado-García, Susana Ivonne Aguirre, Carlos Javier Ortiz, Ana Citlalli Díaz-Leal, Veronica Benavidez, Enrique Peinado, José René Blanco

**Affiliations:** 1 Faculty of Physical Culture Sciences, Autonomous University of Chihuahua, Chihuahua 31000, Mexico; mornelas@uach.mx (M.O.); siaguirre@uach.mx (S.I.A.); cortiz@uach.mx (C.J.O.); ebenavides@uach.mx (V.B.); 2 Hospital Regional Dr. Valentín Gómez Farías ISSSTE, Zapopan 45100, Mexico; citlallidiazmd@gmail.com

**Keywords:** resilience, factorial structure, psychological well-being, sex

## Abstract

Mental health is currently highly relevant in society and one of the factors that could contribute to its improvement is psychological well-being, hence the importance of conducting studies that focus on analyzing variables that predict psychological well-being. Therefore, the goal of this research is to use models of structural equations to analyze the relationships among the variables of sex and resilience for psychological well-being. The total sample was 1190 Mexican university students, with an average age of 20.66 years (SD = 1.89). The results indicate that the resilience factors (strength and confidence, family support, and social support) are the variables with the greatest explanatory power on psychological well-being. It also highlights the mediating capacity of the strength and confidence factor between the other two resilience factors (family support, social support) and perceived psychological well-being. The implications of the study are that sex and two of the dimensions of resilience (family support and social support) show an indirect and positive effect on the perception of psychological well-being through the strength and confidence factor. Therefore, when implementing interventions to improve psychological well-being, these factors should be considered in order to have a greater positive impact on the population that is being studied. Future research should replicate these findings in larger samples.

## 1. Introduction

It is well known that one of the main concerns of human beings lies not only in health but also in finding ways to preserve it (Vasyl, 2018). Individuals, communities and societies around the globe are concerned with mental health, which is determined by multiple socioeconomic (Doran & Irina, 2020; Balon et al., 2016), biological and environmental factors (Lambrette, 2024); mental health and well-being are fundamental for our collective and individual capacity to think, express feelings, interact with others and enjoy life (Salud, 2022), and in recent years, the focus on prevention and mental health care has changed from treatment and prevention only to improving the positive aspects of mental health (Duncan et al., 2021; Schouler-Ocak, 2023).

Since ancient times, human beings have given great importance to their well-being and overall happiness. Efforts to study these concepts go back to Hellenic philosophy; a satisfying life remains a complex matter, such that it has not been completely clarified (Hanying, 2023).

Psychological well-being or eudaimonia emphasizes self-realization and growth, the quality of connections with others, self-knowledge, life management and progressing at people’s own pace (Ryff, 2023), that is, personal development, as well as the ability to create meaning in the face of adversity (Ryff, 2016). This idea (i.e., eudaimonic well-being) includes the concept of a good life at a secondary level and in a personal and subjective manner (Kiaei & Reio, 2014). it is possible to improve psychological well-being through behavioral interventions (Schouler-Ocak, 2023). In this sense, it is important to know the development trajectories of psychological well-being dimensions in order to design interventions that promote mental health and thus achieve a better adaptation to the changes that occur during the life cycle, as well as an optimal execution of the strategies according to the stage lived and considering the characteristics of each person (Mayordomo et al., 2016).

On the other hand, resilience, a relatively new construct associated with the field of psychology, refers to the factors that favor an individual’s ability to overcome adversity in a healthy way (Babić et al., 2020). Resilience is not invulnerability or impermeability to stress (Shackleton et al., 2024), nor is it an intervention technique. Resilience is the ability to endure and defeat adversity, with change and personal self-improvement (Wu, 2023); resilience is the person’s effective coping in the face of adversity, which not only depends on the situation and the subject but also on the environment and their interaction with it (Shackleton et al., 2024). Thus, the resilient person is prepared to face contingencies and emerge stronger (Trejos-Herrera et al., 2023). The resilient personality is made up of three elements that make it possible for resilient responses to be provided in the face of adversity: intrapsychic strengths, skills for action and competencies, and buffer responses (Puig & Rubio, 2013). Many studies have assessed the association between mental health and resilience; there are several investigations in which resilience has been related to psychological well-being, finding a positive correlation between these variables (Cejudo et al., 2016; Guil Bozal et al., 2016; Shackleton et al., 2024). Resilience is therefore a key point in the development of people’s well-being and health, since if resilient factors and resilient capacity are increased, this could help prevent pathologies as well as improve well-being (Ovejero Bruna et al., 2016; Shackleton et al., 2024); resilience research has studied it as a process by which resources protect against the negative impact of stressors to produce positive results (Shackleton et al., 2024).

In addition to the socio-economic, biological and environmental factors that influence mental health, physical activity has been increasingly recognized as a significant contributor to psychological well-being. Research has consistently shown that engaging in regular physical activity can enhance mental health by reducing symptoms of anxiety and depression, improving mood and increasing overall psychological resilience (Mammen & Faulkner, 2013; Schuch et al., 2018). Physical activity is associated with the release of endorphins and other neurochemicals that promote a sense of well-being, while also providing opportunities for social interaction and stress relief (Craft & Perna, 2004). Furthermore, studies have demonstrated that physical activity can improve self-esteem, cognitive function and emotional regulation, all of which are critical components of psychological well-being (Biddle & Asare, 2011; Fox, 1999). Given the growing evidence supporting the positive impact of physical activity on mental health, it is essential to consider its role in interventions aimed at enhancing psychological well-being, particularly among university students who may face high levels of academic stress and emotional challenges.

Arrogante et al. (2015) proposed a structural model in which resilience was a precursor factor in coping, determining the psychological well-being of nursing, concluding that resilience is an inherent characteristic of nursing personnel, that strategies focused on commitment to the stressful situations determine perceived psychological well-being, and that resilience and more adaptive coping strategies constitute two personal resources that determine psychological well-being.

According to Ravera et al. (2016), gender plays a key role in creating resilience, as well as in adaptation pathways.

There are several studies in which it has been found that there are statistically significant differences in resilience based on gender (Ortega et al., 2017; Romero et al., 2015); However, Karmalkar and Vaidya (2018) did not find differences in resilience according to the gender variable.

In their research, Soysa and Wilcomb (2015) found that female university students reported lower scores on assessments of psychological health in contrast to their male counter-parts; they also reported that self-efficacy and gender are predictors of well-being. Likewise, Akhter (2015), in a study of 100 students, reported statistically significant differences by gender in the levels of psychological well-being, which indicates that there is a difference in psychological well-being between men and women.

The following are the studies carried out with students from Ghana, Turkey, Australia, India and Spain on relationships between resilience, psychological well-being and other variables; it is worth mentioning that in the Mexican context, no research was found in this regard.

In a study conducted by Cole et al. (2015) with 431 undergraduate university participants in Ghana, the researchers examined ego resilience and mindfulness and found that these factors cushioned the adverse effects of academic stress on the mental health of university students. An investigation carried out in Turkey with 309 university students by Malkoç and Yalçın (2015) assessed the association between resilience, social support, coping and psychological well-being; their findings showed that resilience, coping, and social support from family, friends and other important people predict psychological well-being, and they also showed that the link between resilience and psychological well-being was partially mediated by coping skills. Aldridge et al. (2016) examined the relationships between the variables of school environment and the feeling of well-being, life satisfaction, ethnic identity, moral identity and resilience among 2122 Australian students; the authors found that the six factors of school environment were related to students’ well-being, indirectly mediated through a sense of ethical and moral identity, resilience, and satisfaction with life, and the teacher support, school connection and affirmative diversity factors had a direct influence.

In India, researchers explored the relationships between optimism, well-being, resilience and perceived stress in 181 university students. The results revealed that optimism has a significant and positive relationship with well-being and resilience; they also found that well-being was positively correlated with resilience and that resilience is also a predictor of well-being (Panchal et al., 2016). Ríos-Risquez et al. (2016) aimed to examine a model of health to determine the extent to which psychological health was explained by resilience and academic burnout in a sample of 113 Spanish university students; the authors found significant relationships between resilience and the three dimensions of academic burnout. The data also showed a significant and negative relationship between resilience and psychological health, measured as the frequency of psychological symptoms. In terms of resilience and academic burnout, nursing students who reported higher resilience also scored higher on their academic effectiveness and, in turn, showed lower scores for emotional burnout and cynicism.

Resilience, psychological well-being and gender (sex) seem to be related, but we consider it important to further the comprehension of these associations. Thus, the purpose of this research was to build a predictive model of psychological well-being based on sex and resilience factors (strength and confidence, family support, social support and structure). To do this, and in the first place, the psychometric properties of the questionnaires used in this study have been assessed in order to guarantee the adequacy of the selected instruments.

Therefore, the goals of this study were, first, to evaluate the psychometric properties of the QEWB (Questionnaire for Eudaimonic Well-Being) designed by Waterman et al. (2010) reviewed by Schutte et al. (2013) and the Mexican Resilience Scale (RESI-M) by Palomar Lever and Gómez Valdez (2010) and, second, to build a predictive model of the perception of psychological well-being in the dimensions sense of purpose and personal expressiveness with purpose based on the variable sex and the resilience factors (strength and confidence, family support, social support and structure).

Tus, our research questions are: What is the most viable and appropriate factor structure for each of the questionnaires used in the study? And What is the model with the most satisfactory structural fit that explains the functional dependency and interrelationships between the variables studied?

### Hypotheses

**Figure 1:**
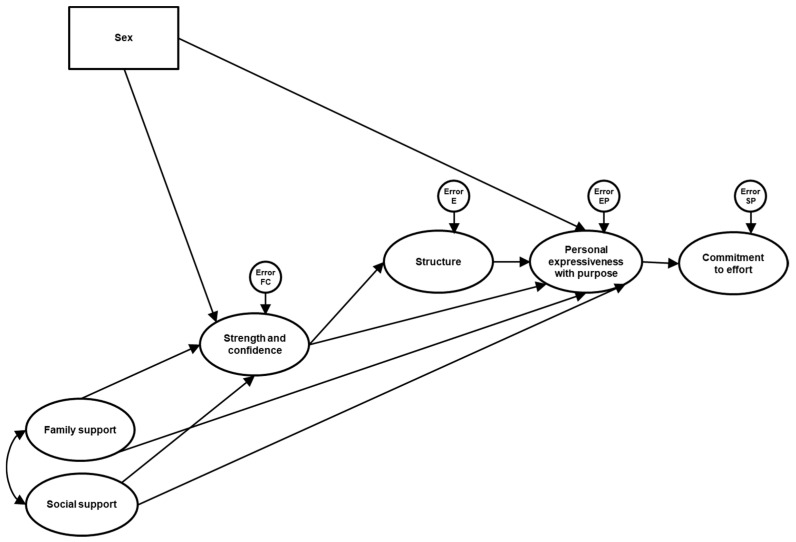
The initial model proposed for psychological well-being based on sex and resilience factors (strength and confidence, family support, social support and structure). Factor indicators are not represented for simplicity in presentation.

From the initially proposed model that integrates the relationships between perceived psychological well-being, sex, and the factors of strength and trust, family support, social support, and resilience structure (Figure 1), the following hypotheses emerge:

Effects of Gender on Psychological Well-Being:

**H1:**  *The variable of sex has an indirect effect on the perception of psychological well-being mediated by the strength and confidence factor and the resilience structure factor.*

**H2:**  *The variable of sex has a direct effect on the perception of psychological well-being.*

Effects of Resilience Factors on Psychological Well-Being:

**H3:**  *The factors of family support, social support, strength and confidence, and structure have a direct effect on the perception of psychological well-being.*

**H4:**  *The factors of family support and social support indirectly influence psychological well-being through the strength and confidence factor and the structure factor.*

**H5:**  *The strength and confidence factor indirectly influences psychological well-being through the structure factor.*

## 2. Materials and Methods

### 2.1. Participants

Of the 1200 invited to participate, only 1190 subjects participated in the study, comprising 628 women (52.77%) and 562 men (47.23%), all university students from Mexico and corresponding to the students who answered all the questionnaires used in this study, and the average age was 20.66 ± 1.89 years.

The research group was selected through convenience sampling, targeting students enrolled in the Human Motricity and Physical Education programs at the Faculty of Physical Culture Sciences. All eligible students were invited to participate, and those who met the inclusion criteria and completed the study protocols were included in the final sample.

The sample was obtained through convenience sampling, trying to cover the representativeness of the different semesters of the degrees offered at the Faculty of Physical Culture Sciences of the 2600 students enrolled.

### 2.2. Instruments and Variables

QEWB (Questionnaire for Eudaimonic Well-Being) is a psychological well-being questionnaire designed by Waterman et al. (2010) and reviewed by Schutte et al. (2013). The QEWB consists of 21 Likert-type items that consistently measure well-being based on the philosophy of eudemonia. Seven of the items are stated in the negative sense. It is characterized by three factors: sense of purpose (α = 0.77), personal expressiveness with purpose (α = 0.73), and commitment to effort (α = 0.61).

In the studied model, only the dimensions of a sense of purpose and personal expressiveness with purpose, factors 1 and 2, resulting from the confirmatory factor analyses, were used.

The Mexican Resilience Scale (RESI-M) by Palomar Lever and Gómez Valdez (2010) is a Likert-type scale and consists of 43 items grouped into five factors: strength and self-confidence (α = 0.92), social competence (α = 0.87), family support (α = 0.87), social support (α = 0.84) and structure (α = 0.79).

In the studied model, four dimensions of the scale were used: strength and confidence, family support, social support and structure resulting from the confirmatory factor analyses.

For sex the value 0 represents women and 1 represents men.

### 2.3. Procedure

This research complies with articles 13, 20 and 21 of the Mexican General Health Law on Health Research; in addition, the study was approved by the Scientific Committee of Research and Postgraduate Studies of the Faculty of Physical Sciences Culture at the Autonomous University of Chihuahua. Thus, students enrolled in both undergraduate degree programs offered by the faculty (Human Motricity and Physical Education) were invited to participate. Two self-report instruments were then applied, QEWB and RESI-M, by means of a personal computer (administrator module of the instrument of the typical performance scale editor), in two sessions that lasted approximately 50 min, in the laboratories or computer centers of the FCCF. Before answering the questionnaires, participants were given instructions to access the instrument, while underscoring that the collected data would remain confidential and that their participation was voluntary. That is, they were told that they could withdraw from the study at any time if they so desired; however, the importance of the research was emphasized.

To ensure transparency in the participant selection process, the study applied specific inclusion and exclusion criteria. Inclusion criteria required participants to be undergraduate students enrolled at the Faculty of Physical Culture Sciences, aged between 18 and 28 years, and willing to provide informed consent. Exclusion criteria included students who did not complete all questionnaires or withdrew from the study at any point. These criteria were implemented to maintain the integrity of the sample and ensure the reliability of the results.

Once the instruments were applied, the results of the scale editor version 2.0 were compiled (Blanco et al., 2013).

For the data analyses, the statistical software SPSS 18.0 and AMOS 21.0 were used.

### 2.4. Data Analyses

#### 2.4.1. Construct Validity and Reliability of the Instruments

To assess the fit of the factorial structure of the instruments with the studied sample, confirmatory factor analyses (CFAs) were performed using the AMOS 21 program (Blanco et al., 2013). In each factor, the variances of the error terms were defined as free criteria, while the factor loads were related to one so that the scale was the same as the items. According to Thompson (2004), when CFA is employed, researchers must verify not only the model fit, but they should compare the fit of various models in order to select the best one.

For the psychological well-being questionnaire, four measurement models were compared: Model 1 (QEWB-3a), a three-factor model according to the distribution proposed by Schutte et al. (2013); for model 2, the items that presented discrimination indices below 0.30 were removed; this model is similar in its factor structure to model 1. Model 3 (QEWB-3c), a three-factor model, was used according to the results of the exploratory factor analysis, and Model 4 (QEWB-3d) was also used, which corresponds to the trifactorial structure of the previous model, eliminating the items that were not sufficiently well explained. And for the resilience scale, two measurement models were compared: Model 1 (RES-IM-5a), a five-factor model according to the original distribution of the items within the questionnaire, and Model 2 (RESIM-5b), which corresponds to a structure of five factors according to the distribution of the model without the items with the lowest saturation in each factor.

Finally, internal consistency for each of the instruments was calculated for the best models, using Cronbach’s Alpha Coefficient (Elosua Oliden & Zumbo, 2008; Nunnally, 1995) and the Omega Coefficient (Revelle & Zinbarg, 2009; Sijtsma, 2009).

#### 2.4.2. Structural Equations Analysis for the Proposed Model

Before using the analysis of structural equations (SEM) to carry out the analysis of the proposed model and to be able to contrast the proposed hypotheses, it was verified that the assumptions underlying this technique were fulfilled, especially those of normality and linearity, for which the values were analyzed for asymmetry and kurtosis and the matrix dispersion graphs of the different variables considered in each model.

Then, from the correlation matrix, SEM was performed using the Maximum Likelihood (ML) estimation method, applying bootstrap resampling procedures for non-normality cases (Byrne, 2013; Kline, 2023) in order to test the set of hypothesized explanatory relationships, even though in AMOS 21.0 the ML is especially robust for possible cases of non-normality, especially if the sample is sufficiently large and the asymmetry and kurtosis values are not extreme (asymmetry < |2| and kurtosis < |7|).

The fit of the models was verified using the Chi-square, the goodness of fit index (GFI), the Standardized Residual Mean Square Root (SRMR) and the Mean Square Error of Approximation (RMSEA) as absolute measures of fit. The Adjusted Goodness Index (AGFI), the Tucker–Lewis Index (TLI), and the Comparative Goodness-of-Fit Index (CFI) were used as measures of incremental fit. The Chi-square ratio on the degrees of freedom (CMIN/DF) and the Akaike Information Criterion (AIC) were used as parsimony fit measures (Blanco et al., 2013; Elosua Oliden & Zumbo, 2008). For the GFI, AGFI, TLI, and CFI, values greater than 0.90 and less than 0.08 were established for the RMSEA and SRMR (Kline, 2023; Sijtsma, 2009).

Once the best model was obtained, the direct or indirect influence among the variables was examined.

## 3. Results

### 3.1. Analysis of the Psychometric Properties of the QEWB Psychological Well-Being Questionnaire

The global results of the CFA (GFI 0.857; RMSEA 0.086; CFI 0.833) for the QEWB-3a model indicate that the measurement model is not acceptable (Table 1).

The set of the three factors of the model QEWB-3a explain approximately 52% of the variance. On the other hand, only 9 of the 21 items have saturations equal to or greater than 0.70 in their expected dimension (items 6, 9, 13, 14, 15, 17, 18, 19 and 20). Furthermore, moderate intercorrelations between the factors were observed, evidencing an adequate discriminant validity between them.

The first model (QEWB-3a) explains 52% of the variance with a three-factor structure that presents moderate discriminant validity, where the items 6, 9, 13, 14, 15, 17, 18, 19 and 20 saturate above 0.70, that is, only 9 of the 21 items (Table 1).

With regard to model (QEWB-3b), the second model tested, the results from the CFA on the fit indices showed a better fit (Table 1); however, it was still not acceptable (GFI 0.882; RMSEA 0.089; CFI 0.861), where the three-factor structure explains 57% of the variance, with a moderate discriminant validity. In addition, only half of the items were saturated over or equal to 0.70 (items 6, 9, 13, 14, 15, 17, 18, 19 and 20).

After the CFA for the third (QEWB-3c) model, optimal fit indices were obtained (GFI 0.971; RMSEA 0.052; CFI 0.972), where the three factors together explain 65% of the variance and only 4 of the 12 items with saturations below the value of 0.70 (items 2, 8, 12, 21) as well, with adequate discriminant validity between the factors.

Finally, the fourth model analyzed (QEWB-3d) showed a three-factor structure for which the CFA indicates that it is better than the previous one and that its fit is optimal (GFI 0.987; RMSEA 0.040; CFI 0.989), with moderate intercorrelations among the dimensions, evidencing an adequate discriminant validity, excluding the items that were not sufficiently well explained. This model explains 70% of the variance, where only 1 (item 12) of the 10 items was saturated below 0.70 on its expected dimension (Table 2).

### 3.2. Analysis of the Psychometric Properties of the Resilience Scale

The global results of the CFA (GFI 0.842; RMSEA 0.059; CFI 0.896) for the RESIM-5a model indicate that the measurement model is not acceptable (Table 3).

In the first five-factor (RESIM-5a) model analyzed, the CFA fit indices showed that the measurement model is not acceptable with GFI values of 0.842; RMSEA 0.059; CFI 0.896 (Table 3); explaining 61% of the variance, with adequate discriminant validity between factors. However, 20 of the 43 items do not load above 0.70 (items 1, 2, 3, 4, 5, 6, 7, 8, 9, 11, 17, 19, 20, 21, 25, 26, 36, 39, 40 and 43).

For the second model RESIM-5b the results from the CFA showed a pentafactorial structure with an optimal fit (GFI 0.973; RMSEA 0.040; CFI 0.984) and an adequate discriminant validity; excluding the items with low saturations, it explained 77% of the variance, where all the items were saturated over 0.70 (Table 4), the only exception being item 19, with 0.68.

### 3.3. Reliability of the Obtained Factors (Internal Consistency)

The resulting factors in the confirmatory factor analyses, in both questionnaires, have, for the most part, internal consistency values above 0.70; in spite of the fact that some factors have a minimum number of items on both instruments, we can conclude that there is an adequate internal consistency (Table 5).

### 3.4. Structural Equation Model to Predict Physical Self-Concept Based on Sex and Physical Health Care

#### 3.4.1. Assessment of the Assumptions Underlying the Analysis of Covariance

Although the values of skew and kurtosis for most variables were ±2.50 and ±7.00, respectively, we did not find multivariate normality as evidenced by a Mardia multivariate index above 70 (Nunnally, 1995).

#### 3.4.2. Overall Fit of the Proposed Models

**Figure 2:**
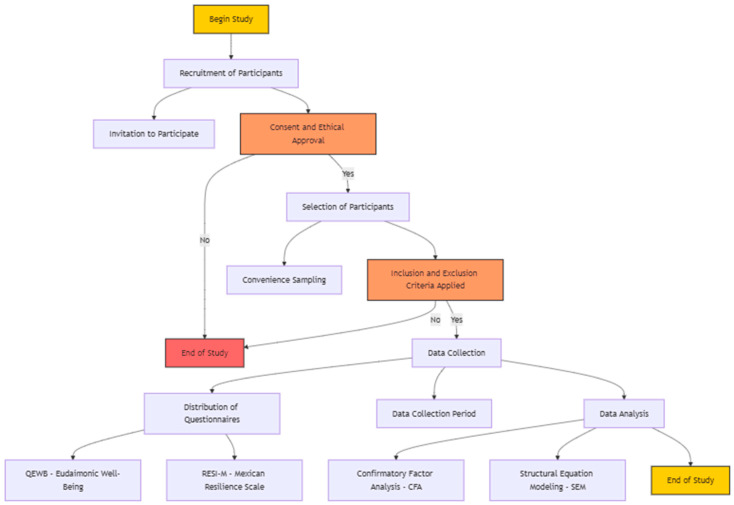
Research process flow chart.

**Figure 3:**
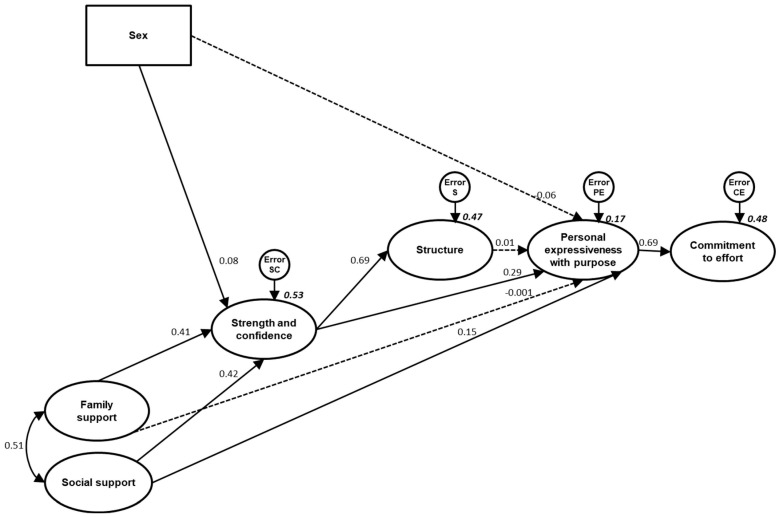
Standardized solution of the initial structural model for psychological well-being based on sex and resilience factors (strength and confidence, family support, social support and structure). All parameters are standardized. Dashed lines represent non-significant paths.

For the initially proposed model, the CFA showed an optimal fit with values of GFI 0.957; RMSEA 0.042; CFI 0.972 (Table 6). However, since three of the expected relationships were not significant (Figure 2), a re-specification of the initial model was performed, eliminating the non-significant relationships: family support > personal expressiveness with purpose, structure > personal expressiveness with purpose, and sex > expressiveness purposeful staff (Figure 3).

After the elimination of the two aforementioned paths, the final model (GFI 0.958; RMSEA 0.045; CFI 0.971) continued to present an optimal fit (Table 6).

**Figure 4:**
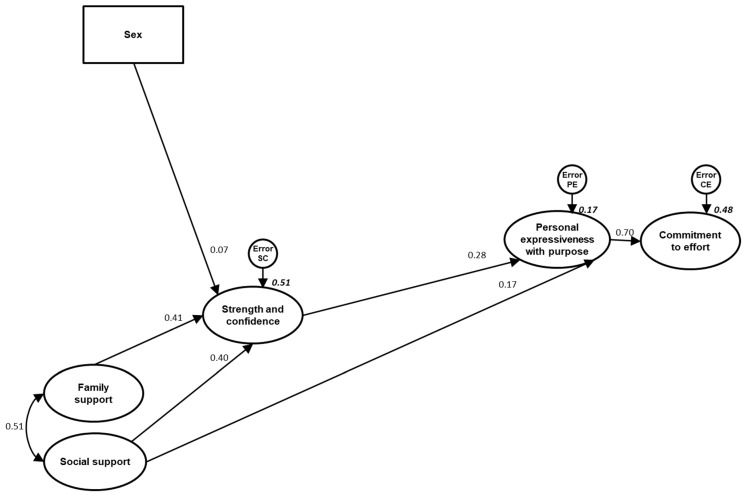
Standardized solution of the final structural model for psychological well-being based on sex and resilience factors (strength and confidence, family support, social support and structure)**.** All parameters are standardized.

Finally, it is observed that both models explain a similar percentage of variance in the criterion variable. Both the initially proposed model and the final model explain approximately 48% of the variance in the perception of physical self-concept in the dimension of physical attractiveness (Figure 3 and Figure 4, respectively).

#### 3.4.3. Assessment of Individual Parameters (Final Model)

By individually analyzing the regression coefficients for each of the pathways proposed in the final model (Figure 4), it was observed that all the proposed relationships obtained a significance at least at a *p* < 0.05 level.

The highest direct effect was the one that produced personal expressiveness with purpose over sense of purpose (β = 0.695, *p* < 0.001), followed by those produced by family support, social support, and sex on strength and confidence. The effect of strength and confidence and social support on personal expressiveness with purpose also stands out. Finally, in Figure 4, it can be seen that all the direct effects are positive.

On the other hand, the results (Table 7) show that social support, family support and sex indirectly influence personal expressiveness with purpose in a significant way. For their part, strength and confidence, social support, family support and sex have an indirect effect on a sense of purpose.

## 4. Discussion

The goals of the present study were first to investigate the psychometric properties of the QEWB (Questionnaire for Eudaimonic Well-Being) designed by Waterman et al. (2010) reviewed by Schutte et al. (2013) and the Mexican Resilience Scale (RESI-M) by Palomar Lever and Gómez Valdez (2010).

The second goal was to build a predictive model of the perception of psychological well-being in the dimensions of a sense of purpose and personal expressiveness with purpose based on the variable sex and the resilience factors (strength and confidence, family support, social support and structure).

Confirmatory factor analyses performed for the QEWB, the fourth model with three factors, explained 70% of the variance and showed adequate intercorrelations among the factors, providing evidence of an adequate discrimination validity among them. The overall results of the CFA for RESIM of the second model tested, corresponding to a pentafactorial structure, explains 77% of the variance, observing discriminant validity. The participants’ social and cultural differences (for university students in the area of physical activity) could underlie the observed discrepancies between the QEWB of Schutte et al. (2013) and the differences found between the model proposed by Palomar Lever and Gómez Valdez (2010).

Regarding the prediction of perceived psychological well-being through sex and resilience, most of the hypotheses raised from the initially proposed model have been fulfilled in such a way that both sex and resilience factors positively predict the perception of psychological well-being in the dimension of personal expressiveness with purpose, and this in turn has a positive direct effect on the sense of purpose dimension of psychological well-being; these results are consistent the findings from other research (Atkinson et al., 2009; Palomar Lever & Gómez Valdez, 2010; Ryff, 2023).

In particular, the variable of sex and the family support and social support factors show a positive indirect effect on the perception of psychological well-being through the strength and confidence factor.

The findings of this study are consistent with previous research that has highlighted the importance of resilience and social support in promoting psychological well-being. For example, Arrogante et al. (2015) found that resilience and adaptive coping strategies were significant predictors of psychological well-being among nursing professionals, which aligns with our results regarding the mediating role of resilience factors. Similarly, Soysa and Wilcomb (2015) reported that self-efficacy and gender were key predictors of well-being, further supporting the indirect effects of sex and resilience observed in our study. Additionally, the positive relationship between family support and psychological well-being echoes findings from Malkoç and Yalçın (2015), who demonstrated that social support from family and friends significantly predicted well-being among university students. These comparisons underscore the robustness of our findings and highlight the universal importance of resilience and social support in shaping psychological well-being across different populations.

One of the contributions of this study is the analysis of the interrelationships among all the variables, since no models were found in the literature with the three variables studied; for example, the study by Arrogante et al. (2015) found that resilience and more adaptive coping strategies constitute two personal resources that determine psychological well-being, as in the research by Soysa and Wilcomb (2015), who reported that self-efficacy and sex are predictors of psychological well-being, results that partially agree with the present study.

### Limitations

The present study has some limitations, among them that the participants are all university students, which limits the possibility of generalizing the results; another limitation is that the measurement instruments are self-reported, which may lead to social desirability bias.

## 5. Conclusions

The results of this study highlight the significant and intricate interplay between the variables analyzed, shedding light on the factors that shape psychological well-being among university students. It was found that sex indirectly influences the perception of psychological well-being through the factors of strength, confidence and structure, while family support and social support also play indirect roles in shaping psychological well-being through similar pathways. Strength and confidence, in turn, directly impact the perception of psychological well-being, with their effects mediated through the structure factor and also operating independently. Additionally, the perception of psychological well-being in the dimension of personal expressiveness with purpose directly contributes to the dimension of a sense of purpose, underscoring the multidimensional nature of well-being.

Overall, the set of variables included in this study explains a substantial portion of university students’ perceived psychological well-being, accounting for 48% of the total variance. This finding underscores the importance of these variables in predicting and understanding psychological well-being, making them crucial targets for educational interventions. Furthermore, the interconnections among these variables demonstrate that they function as a cohesive system rather than in isolation. This intricate network of relationships highlights the necessity of integrating strategies for optimizing and fostering psychological well-being into educational curricula, ensuring a more comprehensive approach to student development.

## Figures and Tables

**Table 1:** Absolute, incremental and parsimony indices for the generated models (QEWB).

	Absolute Indices				Incremental Indices			Parsimony Indices	
Model	χ^2^	GFI	RMSEA	SRMR	AGFI	TLI	CFI	CMIN/DF	AIC
QEWB-3a	1805.63 *	0.857	0.086	0.087	0.822	0.812	0.833	9.708	1895.636
QEWB-3b	1369.63 *	0.882	0.089	0.081	0.847	0.839	0.861	10.376	1447.634
QEWB-3c	217.45 *	0.971	0.052	0.031	0.955	0.964	0.972	4.264	271.455
QEWB-3d	83.01 *	0.987	0.040	0.021	0.974	0.983	0.989	2.862	135.011

Note: * *p* < 0.05. χ^2^ = Chi-square, GFI = goodness of fit index, SRMR = Standardized Residual Mean Square Root, RMSEA = Mean Square Error of Approximation, AGFI = Adjusted Goodness Index, TLI = Tucker–Lewis Index, CFI = Comparative Goodness-of-Fit Index, CMIN/DF = Chi-square ratio on the degrees of freedom, AIC = Akaike Information Criterion.

**Table 2:** CFA standardized solutions for the QEWB-3d Model.

Item	F1	F2	F3
Factor Loadings			
2 I believe I have discovered who I really am	0.70		
9 I can say that I have found my purpose in life	0.77		
13 I believe it is important to know that what I do is in accordance with the goals that are worth achieving		0.79	
14 In general I know what I have to do, because there are certain actions that to me seem correct		0.80	
15 When I get involved in activities that involve my best potentials, I have the sensation of being truly alive		0.75	
17 I find that many of the things I do represent me as the person I am		0.76	
18 It is important for me to feel satisfied with the activities in which I am involved		0.79	
12 I don’t understand why some people work so much on the things they do			0.61
19 If something is very difficult, it is probably not worth doing			0.79
20 I find it difficult to invest a lot on the things I do			0.74
Factor Correlations			
F1	-		
F2	0.68	-	
F3	0.27	0.48	-

Note: F1 = sense of purpose, F2 = personal expressiveness with purpose, F3 = commitment to effort.

**Table 3:** Absolute, incremental and parsimony indices for the generated models (RESI-M).

	Absolute Indices				Incremental Indices			Parsimony Indices	
Model	χ^2^	GFI	RMSEA	SRMR	AGFI	TLI	CFI	CMIN/DF	AIC
RESIM-5a	4417.632 *	0.842	0.059	0.048	0.824	0.890	0.896	5.197	4609.632
RESIM-5b	273.774 *	0.973	0.040	0.028	0.961	0.980	0.984	2.912	357.774

Note: * *p* < 0.05. χ^2^ = Chi-square, GFI = goodness of fit index, SRMR = Standardized Residual Mean Square Root, RMSEA = Mean Square Error of Approximation, AGFI = Adjusted Goodness Index, TLI = Tucker–Lewis Index, CFI = Comparative Goodness-of-Fit Index, CMIN/DF = Chi-square ratio on the degrees of freedom, AIC = Akaike Information Criterion.

**Table 4:** Confirmatory factor analysis standardized solutions for the RESIM-5b Model.

Item	F1	F2	F3	F4	F5
Factor loadings					
10 The confidence I have in myself allows me to overcome difficult moments	0.76				
12 I know how to achieve my goals	0.79				
13 Whatever happens I find a solution to my problems	0.78				
19 When I am not well, I know better times will come	0.68				
23 It is easy for me to have a good conversation topic		0.70			
24 I easily adapt to new situations		0.83			
27 I know how to begin a conversation		0.71			
28 I have a good relationship with my family			0.80		
30 In our family we are loyal among ourselves			0.84		
31 In our family we enjoy doing activities together			0.80		
32 Even in difficult moments, our family has an optimistic attitude towards the future			0.85		
35 I have friends who support me				0.87	
37 I have friends who encourage me				0.89	
38 I have friends who value my abilities				0.87	
41 I plan my activities even in difficult moments					0.78
42 I establish goals even in difficult moments					0.90
Factor Correlations					
F1	-				
F2	0.86	-			
F3	0.62	0.58	-		
F4	0.61	0.61	0.51	-	
F5	0.68	0.65	0.44	0.51	-

Note: F1 = strength and confidence, F2 = social competence, F3 = family support, F4 = social support, F5 = structure.

**Table 5:** Omega and alpha coefficients for the obtained factors.

Factor	Ω	α
Psychological well-being		
Sense of purpose	0.702	0.697
Personal expressiveness with purpose	0.885	0.882
Commitment to effort	0.759	0.758
Resilience		
Strength and confidence	0.840	0.835
Social competence	0.792	0.822
Family support	0.893	0.888
Social support	0.909	0.907
Structure	0.829	0.818

**Table 6:** Absolute, incremental and parsimony indices of the initial and final models for psychological well-being based on sex and resilience factors (strength and confidence, family support, social support and structure).

	Absolute Indices				Incremental Indices			Parsimony Indices	
Model	χ^2^	GFI	RMSEA	SRMR	AGFI	TLI	CFI	CMIN/DF	AIC
Initial	555.398 *	0.957	0.042	0.0558	0.944	0.966	0.972	3.138	663.398
Final	489.962 *	0.958	0.045	0.0560	0.944	0.966	0.971	3.403	581.962

Note: * *p* < 0.05.

**Table 7:** Standardized direct and indirect effects between the variables considered in the final structural model for psychological well-being based on sex and resilience factors (strength and confidence, family support, social support and structure).

		Sex	Family Support	Social Support	Strength and Confidence	Personal Expressiveness with Purpose
Strength and confidence	Direct	0.072	0.413	0.403		
	Indirect					
Personal expressiveness with purpose	Direct			0.170	0.283	
	Indirect	0.020	0.117	0.114		
Sense of purpose	Direct					0.695
	Indirect	0.014	0.081	0.198	0.197	

## Data Availability

Data is available upon request from the correspondence author.
